# Long-Term Monitoring of Microsporidia, *Cryptosporidium* and *Giardia* Infections in Western Lowland Gorillas (*Gorilla gorilla gorilla*) at Different Stages of Habituation in Dzanga Sangha Protected Areas, Central African Republic

**DOI:** 10.1371/journal.pone.0071840

**Published:** 2013-08-07

**Authors:** Bohumil Sak, Klara J. Petrzelkova, Dana Kvetonova, Anna Mynarova, Kathryn A. Shutt, Katerina Pomajbikova, Barbora Kalousova, David Modry, Julio Benavides, Angelique Todd, Martin Kvac

**Affiliations:** 1 Institute of Parasitology, Biology Centre of the Academy of Sciences of the Czech Republic, České Budějovice, Czech Republic; 2 Institute of Vertebrate Biology, Academy of Sciences of the Czech Republic, Brno, Czech Republic; 3 Liberec Zoo, Liberec, Czech Republic; 4 Department of Pathology and Parasitology, Faculty of Veterinary Medicine, University of Veterinary and Pharmaceutical Sciences, Brno, Czech Republic; 5 Faculty of Science, University of South Bohemia in České Budějovice, Czech Republic; 6 Department of Anthropology, Durham University, Durham, United Kingdom; 7 Department of Botany and Zoology, Masaryk University, Brno, Czech Republic; 8 CEITEC - Central European Institute of Technology, University of Veterinary and Pharmaceutical Sciences, Brno, Czech Republic; 9 Department of Ecology, Montana State University, Bozeman, Montana, United States of America; 10 CNRS-Institut des Sciences de l'Evolution de Montpellier, Université de Montpellier II, Montpellier, France; 11 WWF, Dzanga Sangha Protected Areas, Bangui, Central African Republic; 12 Faculty of Agriculture, University of South Bohemia in České Budějovice, Czech Republic; Technion-Israel Institute of Technology Haifa 32000 Israel., Israel

## Abstract

**Background:**

Infectious diseases pose one of the greatest threats to endangered species, and a risk of gastrointestinal parasite transmission from humans to wildlife has always been considered as a major concern of tourism. Increased anthropogenic impact on primate populations may result in general changes in communities of their parasites, and also in a direct exchange of parasites between humans and primates.

**Aims:**

To evaluate the impact of close contact with humans on the occurrence of potentially zoonotic protists in great apes, we conducted a long-term monitoring of microsporidia, *Cryptosporidium* and *Giardia* infections in western lowland gorillas at different stages of the habituation process, humans, and other wildlife in Dzanga-Sangha Protected Areas in the Central African Republic.

**Results:**

We detected *Encephalitozoon cuniculi* genotypes I and II (7.5%), *Enterocytozoon bieneusi* genotype D and three novel genotypes (gorilla 1–3) (4.0%), *Giardia intestinalis* subgroup A II (2.0%) and *Cryptosporidium bovis* (0.5%) in gorillas, whereas in humans we found only *G. intestinalis* subgroup A II (2.1%). In other wild and domestic animals we recorded *E. cuniculi* genotypes I and II (2.1%), *G. intestinalis* assemblage E (0.5%) and *C. muris* TS03 (0.5%).

**Conclusion:**

Due to the non-specificity of *E. cuniculi* genotypes we conclude that detection of the exact source of *E. cuniculi* infection is problematic. As *Giardia intestinalis* was recorded primarily in gorilla groups with closer human contact, we suggest that human-gorilla transmission has occurred. We call attention to a potentially negative impact of habituation on selected pathogens which might occur as a result of the more frequent presence of humans in the vicinity of both gorillas under habituation and habituated gorillas, rather than as a consequence of the close contact with humans, which might be a more traditional assumption. We encourage to observe the sections concerning hygiene from the IUCN best practice guidelines for all sites where increased human-gorilla contact occurs.

## Introduction

Over 1,000 mammalian species are red-listed by IUCN, as Critically Endangered, Endangered or Vulnerable as a result of poaching, bushmeat hunting and trade, invasive species, disease and habitat encroachment. Funds needed to combat these threats are lacking; consequently conservation efforts based on non-consumptive use of natural resources such as wildlife tourism have become a widespread source of funding [1]–[3]. Whilst wildlife tourism currently contributes to the survival of many endangered mammals [4], a growing body of research has also highlighted significant detrimental impacts on focal animals and habitats, of which the long-term effects on wildlife remain largely unquantified. Infectious diseases pose one of the greatest threats to endangered species [5], [6] as anthropogenic disturbance and habitat alteration significantly increase the risk of disease transmission and spread amongst wildlife populations [7], [8].

Several animal species have been habituated to human presence for the purposes of wildlife viewing [9] but great apes in particular are a popular focus of habituation efforts [10]–[13]. During the habituation process animals become accustomed to human presence and are thought eventually to accept a human observer as a neutral element in their environment [14].

The western lowland gorilla is classified as a critically endangered species as a result of hunting, diseases, habitat loss and degradation and potential impacts of climate change [15]. Mountain gorilla programs have demonstrated the potential for gorilla conservation via tourism [16], [17]. However, the consequences of increasing human-gorilla contacts in habituated groups (and even in adjacent unhabituated ones), and therefore the long-term sustainability of these programs, remain poorly understood [18]–[23]. A risk of pathogen transmission from humans to gorillas is a major concern of gorilla tourism [18], [24], [25]. For example, chronic stress caused by increased contact with humans may reduce immunity and therefore increase gorillas’ susceptibility to novel and human-borne diseases [20], [26]–[28]. This situation calls for a thorough assessment of the impact of human presence.

Gastrointestinal parasites are commonly listed as important risk factors in primate conservation efforts [20], [29]–[31]. An increased anthropogenic impact on primate populations may result in general changes in communities of their parasites, and also in a direct exchange of parasites between humans and primates. However, besides common speculations, there remains little direct evidence for parasite transmission between humans and wild great apes, even in the habituated communities, because the presence of a parasite in both humans and apes does not necessarily imply pathogen transmission. Thus, understanding the epidemiology and zoonotic transmission of parasites in the human-ape interface calls for a broad use of molecular genotyping and subtyping tools [31]–[34].

Microsporidia, *Cryptosporidium* spp., and *Giardia* spp. are protists with environmentally resistant stages, capable of infecting a range of vertebrates and are some of the most common intestinal parasites of humans [35]–[37]. Despite a plethora of studies dealing with various aspects of parasitic infections in captive and free ranging great apes, those addressing the diversity and prevalence of intestinal microsporidia, *Cryptosporidium* and *Giardia* are limited [38]–[41]. In primates, it has been repeatedly hypothesized that the presence and/or increased prevalence of microsporidia, *Cryptosporidium,* and *Giardia* results from increased contact with humans and/or livestock [29], [32], [41]–[44].

To better understand the impact of habituation and close contact with humans on the occurrence of potentially zoonotic protists in great apes, we conducted a long-term study in wild western lowland gorillas (*Gorilla gorilla gorilla*), humans, and other wildlife in Dzanga-Sangha Protected Areas (DSPA) in the Central African Republic, one of the first sites where the lowland gorillas were ever habituated. Using molecular methods, we (i) address the diversity of microsporidia (*Encephalitozoon* and *Enterocytozoon*), *Cryptosporidium*, and *Giardia* in gorillas and other hosts; (ii) assess the potential for cross-transmission between these subject groups; and (iii) analyse the impact of human-ape contact on the occurrence of these pathogens in several groups of gorillas at different stages of the habituation process.

## Materials and Methods

### Ethics Statement

The research complied with the legal requirements of the Central African Republic and adhered to the research protocol of DSPA. All sample collection from humans was approved by the Anthropology Department Research Ethics and Data Protection Committee; University of Durham, U.K. Only a verbal non-recorded consent was obtained from all examined persons and the samples were anonymized. We also obtained a verbal non-recorded permission from the owners of domestic animals to collect animal’s faecal samples. The IRB approved this consent procedure. The collection of faecal samples from gorillas, other wildlife and livestock was noninvasive and did not cause any observable distress to the animals.

We would like to thank the government of the Central African Republic and the World Wildlife Fund for granting permission to conduct our research in the Central African Republic; the Ministre de l'Education Nationale, de l'Alphabetisation, de l'Enseignement Superieur, et de la Recherche for providing research permits; and the Primate Habituation Programme for providing logistical support in the field.

### Study site

The Dzanga-Sangha Protected Areas (DSPA) in the Central African Republic (CAR) includes areas of different protected status: the strictly protected Dzanga- Ndoki National Park (1,222 km^2^) with restricted human access and Dzanga Sangha Dense Forest Special Reserve (3,159 km^2^), a multiple-use zone in which human activities are controlled [45]. The human population density in DSPA is low, estimated at 6,000 (approx. 1 person per km^2^) [45], [46]. The forest can be classified as a mix of habitats including secondary forest, but primary forest is also common. The abundance of wildlife was described first by Carroll [47] and updated for primates by Blom et al. [45] and recently by Remis and Jost Robinson [46]. Rainfall averages 1,400 mm/year, dry months typically occur between December and February; the rest of the year has a long rainy season with a relatively drier period between June and July [46], [47]. A portion of DSPA was selectively logged at low intensity in the past.

Our sampling was carried out in two main study sites in the Dzanga Sector of the National Park: (i) Bai Hokou (2°50'N, 16°28'E) and (ii) Mongambe (2°55'N, 16°23'E) and their surroundings. Both Mongambe and Bai Hokou are permanent Primate Habituation Programme (PHP) research camps with approximately 13 trackers, 2–3 research assistants and 1–3 researchers/volunteers in each camp (see 2.3. for a detail description of human contact with gorillas). The largest human settlement, Bayanga, is approximately 30 km from these sites (approx. 3,925 inhabitants) [48].

### Studied gorilla groups

#### Habituated gorillas

Habituation of Makumba group at Bai Hokou began in 2000 and the group was opened to tourists in September 2004. The group composition varied over the four years due to emigrations and birth of new infants, resulting in 14 members in 2007 and reduced to 10 members in 2010. Habituation of Mayele group at Mongambe started in 2005 and the group was opened to tourists at the beginning of 2010, but the number of tourists was significantly lower (2010: 114) in comparison with Makumba group (2007: 391; 2008: 409; 2009: 343; 2010: 257). Both Makumba and Mayele groups are followed daily by two teams of local BaAka trackers (2–3 per team), Bantu assistants and/or foreign researchers/volunteers (1–2 per team), and irregularly visited by tourists. Observers aim to maintain a minimum distance of 7 meters or more from the gorillas as specified by gorilla tourism regulations of the PHP. The regulations also restrict viewing of gorillas to a maximum of 60 min for those joining the tracking team and tourist groups are limited to two per day, with a maximum of three tourists per group.

#### Gorilla groups under habituation

Habituation of two new groups at Mongambe and Bai Hokou started in 2008. One or two teams per day usually consisting of two BaAka trackers and one researcher/Bantu assistant walk along forest trails in the known home ranges of the gorilla groups targeted for habituation until fresh tracks of the groups are found. They then follow the tracks until gorillas are located. At the beginning of the habituation process, contacts of the specifically targeted group and consequently any contacts can be highly sporadic (lasting several minutes at best). Furthermore, although ideally one to two teams are deployed on a daily basis, the shortage of trackers can often result in days when no teams are available for habituation efforts. Nevertheless by 2009, habituation teams at the Mongambe camp were able to intermittently follow the trail of a group which was named Wonga (10–15 members). Similarly, at Bai Hokou, a single group named Mata with approximately 8 members was followed from late 2009. Attempts were made to make no more than one contact per day per group. In 2009 habituation teams were searching for groups on average 13 days per month, they were able to locate traces of the group on 7 days per month and made approximately 4 contacts per month. In 2010 teams were searching for groups on average 27 days per month, they were able to more consistently locate traces on 16 days per month and made approximately 6 contacts per month.

#### Unhabituated groups

Several completely unhabituated groups and solitary males range in the Dzanga sector of DSPA. They can be accidentally encountered by the habituation teams; however, there are no systematic attempts to locate them.

### Sample collection

Faecal samples from gorilla groups were collected yearly during November and December from 2007 to 2010 with the largest sample set obtained in 2009 (Table 1). In Makumba group, each member could be individually recognized and preferably individual samples were collected. We aimed to collect at least one sample per individual every year, but some individuals were sampled twice or more, especially in 2009. In Mayele group unidentified samples were collected from night nests during the entire study period because of the unreliability of recognizing all individuals. No samples from Mayele group were obtained in 2008. Samples from groups under habituation and unhabituated groups were obtained in 2009 and 2010 from night nests and from trails during tracking. Samples from unhabituated gorillas were opportunistically collected outside of the ranges of habituated groups and groups under habituation by a researcher and a BaAka tracker team on specific patrols. The Mayele group and groups under habituation were sampled repeatedly every few days, but for unhabituated groups it was impossible to reliably get samples from the same group more than once. In 2009, we obtained unidentified nest samples from four unhabituated groups and in 2010 from unhabituated three groups and one solitary male.

**Table 1:** *Enterocytozoon bieneusi*, *Encephalitozoon cuniculi*, *Cryptosporidium* spp. and *Giardia intestinalis* infection in wild western lowland gorillas (*Gorilla gorilla gorilla*) under different levels of human contact.

Human contact	Groups	Year	n1	n2	Positive samples				
					*Encephalitozoon* spp.	*Entereocytozoon bieneusi*	*Cryptosporidium* spp.	*Giardia intestinalis*	
**unhabituated**	1	2009	8	8	-	-	-	-	
	2	2009	7	7	-	-	-	-	
	3	2009	3	3	-	-	-	-	
	4	2009	2	2	-	-	-	-	
	5	2010	3	3	-	1× gorilla 1	-	-	
	6	2010	6	6	-	-	-	-	
	7	2010	1	1	-	-	-	-	
	8	2010	1	1	1× EC II	-	-	-	
**under habituation**	Wonga	2009	14	14	1× EC I; 1× EC II	1× D; 1× gorilla 3	-	-	
		2010	5	5	-	-	-	-	
	Mata	2009	24	8	2× EC I	-	-	1× A	
		2010	4	4	-	-	-	-	
**fully habituated**	Makumba	2007	21	14	2× EC I	-	1× *C. bovis*	-	
		2008	10	10	2× EC I	-	-	1× A	
		2009	33	11	4× EC I	1× D	-	2× A	
		2010	13	8	-	1× gorilla 3	-	-	
	Mayele^ a^	2007	11	11	-	-	-	-	
		2009	24	11	1× EC I	1× D; 1× gorilla 2	-	-	
		2010	11	11	1× EC II	1× gorilla 2	-	-	
			**201**	**89**	**15**	**8**	**1**	**4**	

**D** =  *E. bieneusi* genotype D; **gorilla 1**  =  *E. bieneusi* genotype gorilla 1; **gorilla 2**  =  *E. bieneusi* genotype gorilla 2; **gorilla 3**  =  *E. bieneusi* genotype gorilla 3; **EC I**  =  *E. cuniculi* genotype I; **EC II**  =  *E. cuniculi* genotype II; **A**  =  *Giardia intestinalis* assemblage A; **n^1^** number of samples; **n^2^** number of animals sampled.

Faecal samples of other wild mammals (for species see Table 2) including habituated agile mangabeys were collected in the same area in 2010. In 2010 we also obtained samples from domestic animals in the Bayanga village - the principal human settlement within DSPA. Human stool samples were collected from ecoguards, PHP gorilla trackers, assistants and researchers during regular health monitoring of the Park employees in 2009­–2010. All faecal samples were immediately preserved in 96% ethanol and later transported to the Institute of Parasitology, Biology Centre of Academy of Sciences of the Czech Republic.

**Table 2:** Enterocytozoon bieneusi, Encephalitozoon cuniculi, Cryptosporidium spp. and Giardia intestinalis infection in humans, wild and domestic animals.

Host	Year	n	Positive samples			
			*Encephalitozoon* spp.	*Entereocytozoon bieneusi*	*Cryptosporidium* spp.	*Giardia intestinalis*
**Humans**	2010	48	-	-	-	1× A
**Other wildlife**						
*Loxodonta africana*	2010	52	-	-	-	-
*Potamochoerus porcus*	2010	23	-	-	-	-
*Hylochoerus meinertzhageni*	2010	2	-	-	-	-
*Cephalophus callipygus*	2010	15	1× EC I	-	-	-
*Cephalophus dorsalis*	2010	12	1× EC I	-	-	-
*Cephalophus monticola*	2010	10	-	-	-	-
*Cephalophus silvicultor*	2010	2	-	-	-	-
*Tragelaphus euryceros*	2010	6	-	-	-	-
*Syncerus caffer*	2010	20	1×EC I; 1× ECII	-	1× *C. muris* TS03	-
*Cercocebus agilis*	2008	6	-	-	-	-
	2009	10	-	-	-	-
	2010	17	-	-	-	-
**Domestic animals**						
*Ovis ammon aries*	2010	2	-	-	-	-
*Capra aegagrus hircus*	2010	9	-	-	-	1× E
*Sus scrofa*	2010	5	-	-	-	-

**n**  =  number of samples; **EC I**  =  *E. cuniculi* genotype I; **EC II**  =  *E. cuniculi* genotype II; **E**  =  *Giardia intestinalis* assemblage E; **A**  =  *Giardia intestinalis* assemblage A.

### DNA extraction, PCR amplification, sequencing and genotyping

The suspension of each faecal sample in alcohol was evaporated overnight at 60°C. A total of 200 mg of faecal samples were homogenized by bead disruption using 0.5 mm glass beads (Biospec Products, Inc., Bartlesville, OK, USA) in a FastPrep®-24 Instrument (MP Biomedicals, CA, USA) at a speed of 5 m/s for 1 min followed by isolation/purification using the QIAamp® DNA Stool Mini Kit in accordance with the manufacturer’s instructions (Qiagen, Hilden, Germany). Purified DNA was stored at –20°C prior to use in PCR. All DNA samples obtained for the study were analysed by polymerase chain reaction (PCR) using sets of specific primers. A nested PCR approach was used to amplify a region of the internal transcribed spacer (ITS) of *Enterocytozoon bieneusi* (∼390 bp) [49], the small ribosomal subunit rRNA gene (SSU) of *Cryptosporidium* spp. (∼ 830 bp) [50], and the triosephosphate isomerase gene (TPI) of *Giardia intestinalis* (∼500 bp) [51]. The following primer sets were used to amplify *Encephalitozoon* spp.: the int580f and int580r primer set for primary PCR analysis [52] and the MSP3 and MSP4 primer set for secondary PCR (∼320 bp) [53]. Secondary PCR products were run on a 2% agarose gel containing 0.2 µg/ml ethidium bromide in 1× TAE buffer at 75 volts for approximately 1 hour. Bands of the predicted size were visualized using a UV light source, cut from the gel, and then extracted using QIAquick Gel Extraction Kit (Qiagen). Gel purified secondary products were sequenced in both directions with an ABI 3130 genetic analyzer (Applied Biosystems, Foster City, CA) using the secondary PCR primers and the BigDye1 Terminator V3.1 cycle sequencing kit (Applied Biosystems, Foster City, California) in 20 µl reactions.

Positive and negative controls were included in each analysis. DNA from *E. intestinalis* spores grown *in vitro* in the Laboratory of Veterinary and Medical Protistology at the Institute of Parasitology of ASCR, from *E. bieneusi* spores of genotype S6 originally isolated from an eastern house mice, from *Cryptosporidium serpentis* originating from a corn snake, and from *Giardia intestinalis* assemblage E originating from a domestic goat were used as positive controls for appropriate PCR. All samples were analysed in duplicates. In case of positive detection the sample was newly re-isolated and the previous finding was independently verified.

### Phylogenetic analyses

The nucleotide sequences of each gene obtained in this study were edited using the ChromasPro 1.5 software (Technelysium, Pty, Ltd.) and were aligned with each other and with reference sequences from GenBank using ClustalX 2.0.6. Alignment adjustments were made manually to remove artificial gaps using BioEdit.

Phylogenetic analyses were performed using the MEGA5 software [54]. Neighbour joining (NJ) trees were constructed. All ambiguous positions were removed for each sequence pair. The reliability of branches in trees was assessed using the bootstrap analysis with 1000 pseudo-replicates, with values above 50% reported. Phylograms were drawn using MEGA5 and were manually adjusted using CorelDrawX5. ITS, SSU, and TPI sequences have been deposited in GenBank under the accession numbers JQ837793-JQ837800, JQ837801-JQ837802 and JQ837803-JQ837808, respectively.

### Statistical analyses

To analyse the effect of habituation on the occurrence of microsporidia in gorillas we fitted the generalized linear mixed model (GLMM) with binomial distributions. For the analyses we randomly chose data from 98 gorillas (2009–2010) with each individual represented by one sample to avoid bias from repeated sampling occurring in habituated animals and especially in unidentified gorillas under habituation and from Mayele group. In gorillas under habituation and of Mayele group we chose nest samples from a single day. Unhabituated gorillas were sampled only once. Animals were classified according to the “group“ (Mayele, Makumba, Wonga, Mata and several unhabituated groups) and “level of human-ape contact“ (habituated, under habituation, unhabituated). The random factor “group“ was nested into the fixed factor “level of human-ape contact“. We employed Fisher exact tests to examine temporal changes in the occurrence of microsporidia in habituated groups (Makumba: 2007, 2008, 2009, 2010; Mayele: 2007, 2009, 2010) and groups under habituation (both groups: 2009, 2010). To avoid bias from repeated sampling in particular years, we decided to randomly choose one sample per individual in the same way as described above. Due to unidentified samples (Mayele group and groups under habituation) and frequent changes in a group composition (Makumba) we could not implement pair tests in this case. We did not perform any similar analyses for *Giardia* and *Cryptosporidium* infections due to a low number of detected cases.

## Results

Out of the total of 201 examined gorilla’s samples 28 (13.9%) were positive for tested parasites. The most frequently detected parasites were *Encephalitozoon* spp. in 15 (7.5%) and *E. bieneusi* in 8 (4.0%) samples respectively. *Giardia intestinalis* was identified in 4 (2.0%) samples and *Cryptosporidium* sp. in a single case (0.5%) (Table 1). We did not detect any differences in the occurrence of microsporidia in habituated, under habituation and unhabituated gorilla groups (GLMM, treatment contrasts given: habituated vs. under habituation z = –0.45, *p* = 0.65; habituated vs. unhabituated z = –1.73, *p* = 0.08; under habituation vs. unhabituated z = –1.37, *p* = 0.17). No changes among years were recorded in the occurrence of microsporidia in any group (Fisher exact tests: Makumba *p* = 0.44, Mayele *p* = 0.33, Mata *p* = 1.0, Wonga *p* = 1.0).

Only one of the 48 human stool samples (2.1%) was positive for *Giardia intestinalis* and none of the other parasites investigated was detected. Out of the total of 191 samples obtained from other wild and domestic animals 6 (3.1%) were positive for the presence of specific DNA of screened parasites, including 4 cases of *Encephalitozoon* spp. (2.1%), one case of *Giardia intestinalis* (0.5%) and one case of *Cryptosporidium* sp. (0.5%) (Table 2).

**Figure 1:**
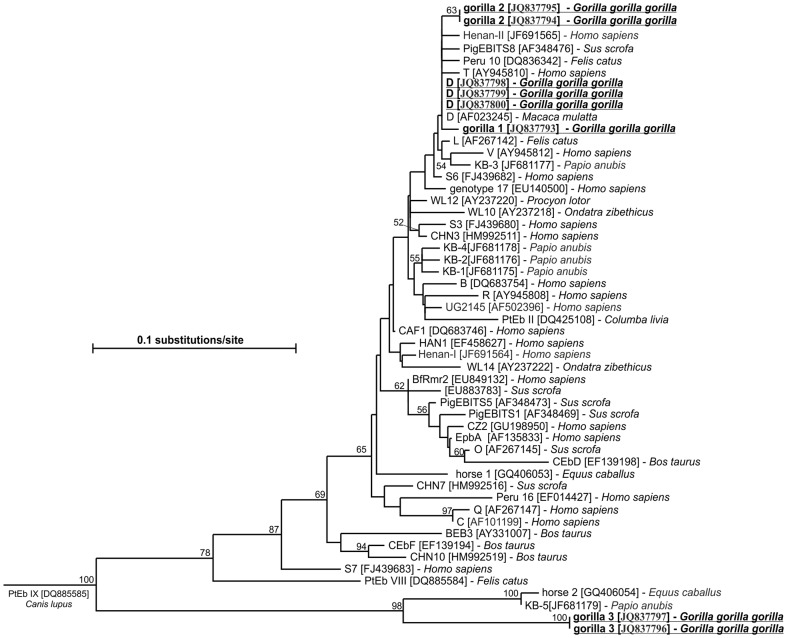
Neighbour-joining tree based on nucleotide sequences of the whole ITS region of Enterocytozoon bieneusi isolates, including our new sequences (underlined). Genotypes previously found in apes and humans are shaded. The host is listed for each sample. Values on branches are percent bootstrapping using 1 000 replicates. The bootstrap proportions greater than 50% are shown on each branch. Nucleotide sequences generated from this study are underlined and are deposited in the GenBank under Accession Nos. JQ837793-JQ837800.

The alignment of the obtained microsporidial ITS sequences with reference sequences showed 100% homology with GenBank-listed species and their genotypes as follows: 13 *E. cuniculi* genotype I (AF338410) and 4 *E. cuniculi* genotype II (GQ422153) (Tables 1 and 2). A phylogenetic analysis of all ITS sequences performed on a multiple alignment that included representatives of *E. bieneusi* genotypes accessible in GenBank revealed in three cases the presence of previously described pathogenic human *E. bieneusi* genotype D (AF101200) in gorillas. Moreover, five sequences of our isolates belonged to three new genotypes. While two new genotypes named gorilla 1 and gorilla 2 matched genotype D closely with 99.1 and 99.5% similarity, a third new genotype named gorilla 3 is probably unique to gorillas (two cases). When ITS nucleotide sequences of gorilla 3 genotype were compared with other sequences in the GenBank database, the closest matches were 93.6% similarity to *E. bieneusi* genotype KB-5 (JF681179) isolated from a captive olive baboon in Kenya and 93.2% similarity to horse 2 genotype (GQ406054) isolated from a domestic horse in Columbia. The global topology of the tree is shown in Fig. 1.

**Figure 2:**
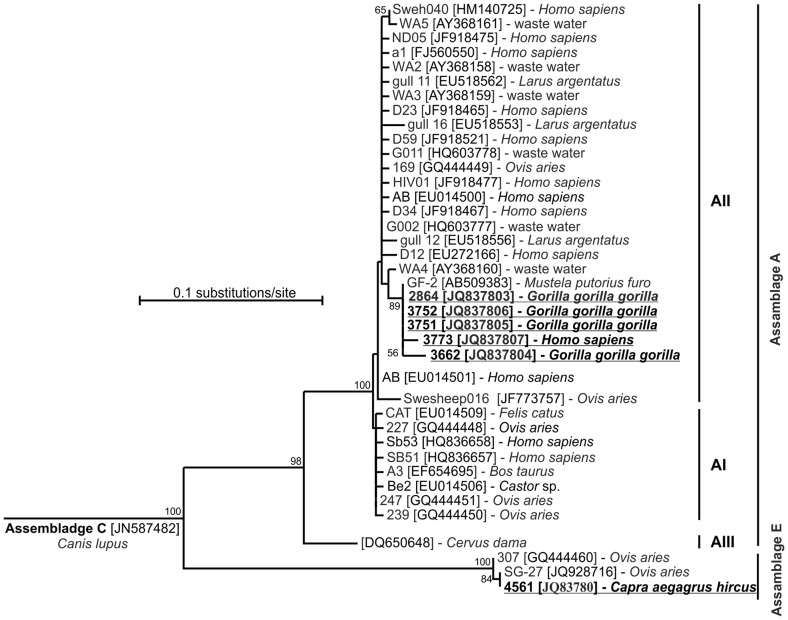
Neighbour-joining tree based on partial nucleotide sequences of the TPI gene of Giardia intestinalis, including our new sequence (underlined). Genotypes previously found in apes and humans are shaded. The host is listed for each sample. Values on branches are percent bootstrapping using 1 000 replicates. The bootstrap proportions greater than 50% are shown on each branch. Nucleotide sequences generated from this study are underlined and are deposited in the GenBank under Accession Nos. JQ837803-JQ837808.

As shown in Fig. 2, on the basis of the phylogenetic tree based on TPI sequences we were able to resolve *G. intestinalis* clades of assemblage A originating from humans and gorillas and assemblage E from a sheep. All of the identified assemblage A sequences belonged to A II subgroup forming a well-supported sister group to an isolate from waste water. Three of the five *G. intestinalis* sequences were identical to GF-2 isolate (AB509383) previously reported from a domestic ferret and another two sequences matched GF-2 isolate most closely, with 99.4–99.6% similarity.

Phylogenetic analyses based on SSU sequences showed that *Cryptosporidium* originating from a habituated gorilla was 100% similar to the *C. bovis* reference sequence listed in GenBank (AY741305). Another *Cryptosporidium* sequence obtained in this study from a forest buffalo was identical to *C. muris* TS03 (EU245043, phylogeny not shown).

A single-species infection was detected in all animals with the exception of one co-infection with *E. bieneusi* genotype D and *G. intestinalis* apparent in a gorilla from Makumba group.

## Discussion

Microsporidia, *Cryptosporidium* spp., and *Giardia* spp. belong to a group of opportunistic pathogens that are notable for cross-species transmission [55], [56] due to their low host specificity. The mechanisms of host specificity remain unknown, but their frequency of crossing the host barrier and becoming zoonotic is increasing [57]–[59].

Results from captive facilities suggest that a close human-ape contact might be important for the occurrence of microsporidia in apes, which may be applicable to natural conditions [41]. Here, we did not detect a significant increase in the occurrence of microsporidia in habituated gorillas compared to gorillas under habituation or unhabituated gorillas, as would be expected based on the increased human-gorilla contact with habituated groups. It is well known that microsporidia are ubiquitous in the environment, and it is therefore likely that any gorilla will be infected with microsporidia during their lifetime [60], [61]. However, the concentration and frequency of spores shed by immunocompetent individuals are very low and sporadic [61]. If considering all data on microsporidia including repeatedly sampled habituated gorillas and gorillas under habituation, there is an indication that these gorillas may shed microsporidia spores into the environment more frequently in comparison with unhabituated gorillas (Table 1), however no statistical comparison was possible. If habituated gorillas and gorillas under habituation experience increased stress – and therefore have reduced immune functions, we suggest that this may lead to a manifestation of chronic infection with microsporidia and therefore greater shedding of spores into the environment. A similar situation has been described in other hosts including humans [61]–[63]. To better test this hypothesis, repeated individual sampling of unhabituated groups and individual sampling of groups under habituation would be necessary to acquire data comparable to that from habituated animals. Advances in genotyping of the individuals based on faecal DNA can help to address this problem and avoid bias resulting from work with unidentified faecal samples, but repeated sampling of completely unhabituated gorillas remains a challenge.

Our results show that microsporidia were commonly present in gorillas and other wildlife of the DSPA, but less frequently in a domestic livestock. The most common causative agents of mammalian microsporidiosis are *Enterocytozoon bieneusi* and microsporidia from the genus *Encephalitozoon* [64]. As of this date, *E. intestinalis* is the only species which has been identified in wild great apes, namely in habituated mountain gorillas in Uganda and also in two persons who shared habitats with them [39]. However, in this study we detected *Encephalitozoon cuniculi* in gorillas, domestic and wild animals and *Enterocytozoon bieneusi* only in gorillas.

We identified *E. cuniculi* genotypes I and II with genotype I as the most prevalent. The same genotypes of *E. cuniculi* have also been detected in captive gorillas and chimpanzees from several zoos and sanctuaries [41]. *E. cuniculi* genotypes are not host-specific and have been identified in a variety of animal hosts such as rodents, lagomorphs, carnivores, human and nonhuman primates, and birds [65]–[67]. Unlike Sak et al. [41], who recorded a high prevalence of *E. cuniculi* genotype I among both animal keepers and apes in the Limbe Wildlife Centre, Cameroon, implying the potential human to ape or *vice versa* transmission, we did not find any microsporidia in humans. However, we detected genotype I in two species of *Cephalophus* and both I and II in forest buffalos. Based on our results we conclude that due to the non-specificity of *E. cuniculi* genotypes and their occurrence in various hosts, detection of the exact source of *E. cuniculi* infection in gorillas is difficult.

To the best of our knowledge, this is the first report of *Enterocytozoon bieneusi* infection in wild great apes. Sequence analyses showed four distinct *E. bieneusi* genotypes including three new ones. Genotype D, which was the most prevalent, has identical ITS sequences to those previously described in humans, including those detected in AIDS patients in Africa and also in a variety of domestic and wild animals including macaques and olive baboons [68], [69]. Sak et al. [41] also reported genotype D in captive chimpanzees from a European zoo and a sanctuary in Kenya. Two of the three new *E. bieneusi* genotypes in gorillas, gorilla 1 and gorilla 2, were genetically related to genotype D. All of these genotypes belong to group 1, which was identified by Thellier and Breton [70], and have a zoonotic potential and public health significance. The new gorilla genotype 3 formed a well-supported branch, located separately in the phylogenetic tree. This genotype clusters with a group of isolates previously found in horses and baboons [70] and may represent a host-adapted genotype in primates.

Our results demonstrate that *Giardia intestinalis* is present at a relatively low prevalence in gorillas, primarily in groups with closer human contact. However, *Giardia* cysts are also intermittently shed into the environment, which, when combined with our sampling limitations, makes the correct detection of positive individuals in unhabituated groups particularly problematic. Graczyk et al. [40] analysed samples from both habituated and unhabituated mountain gorillas and found *Giardia intestinalis* only in two unhabituated gorillas, suggesting transmission among people, cattle and mountain gorillas. Unlike Graczyk et al. [40], we were able to classify *Giardia* assemblages and their subtypes more accurately, and demonstrated the presence of two assemblages by phylogenetic analyses based on TPI gene sequences. While the isolate originating from a domestic goat belonged to assemblage E, all isolates obtained from gorillas and a human belonged to assemblage A. Among *G. intestinalis* assemblages, assemblage A has the broadest host specificity infecting humans, livestock and wildlife including primates (gorillas, southern brown howler monkeys, etc.) [36]. The two most common sub-assemblages of assemblage A, subgroups A I and A II, appear to differ in host preference [71], [72]. Humans are more commonly infected with subgroup A II, whereas animals are frequently infected with subgroup A I [36]. The fact that the detected assemblages in gorillas and in a human belong to subgroup A II may suggest that human-gorilla transmission has occurred. This implies a potential negative impact of habituation which might occur as a result of the more frequent presence of humans in the ranges of habituated and gorillas under habituation. Humans can contaminate the environment with *Giardia* cysts and subsequently gorillas can be infected by these cysts via the faecal-oral route. However, due to a low number of detected cases in both gorillas and humans, and taking into consideration the intermittent cyst shedding with our known sampling limitations, we suggest that further study of this topic is necessary to confirm this suggestion.

Only one previous study detected the presence of zoonotic *Cryptosporidium parvum* in wild great apes, namely in mountain gorillas [38]. Most of the other studies concerned with cryptosporidia in great apes and even in other primates did not discriminate species/genotypes [31], [43], [44], [73]–[75]. In the present study we detected only one *C. bovis* isolate originating from a habituated gorilla. *Cryptosporidium bovis* is considered to have adapted itself to domestic ruminants (cattle and sheep) [76], [77] or African buffalos [78] and no transmission between specific host and primates has been reported to date [79], however, it is possible that under certain conditions this host-specific *Cryptosporidium* can be transmitted between species [80], [81]. Although we did not find *C. bovis* in domestic or wild ruminants in the DSPA, we still suggest that cross-transmission of *Cryptosporidium* is more likely to occur among gorillas and wild ruminants in the DSPA in case that livestock do not enter or approach the Park. Alternatively, the detection of a single case of *C. bovis* in gorillas could also be explained by simple passage of oocysts through the digestive tract without any ongoing infection.

To our knowledge, this is the first study providing detailed molecular information about *Cryptosporidium*, microsporidia and *Giardia* infections in wild western lowland gorillas and great apes in general. These results document a baseline occurrence of these parasites in gorilla groups with various levels of human contact and suggest that well-managed ecotourism and research activities may result in a minimal impact on microsporidia, *Cryptosporidium* and *Giardia* infections in wild western lowland gorillas. We specially urge other sites to follow the best practice guidelines for tourism [82] when working with unhabituated gorillas or gorillas under habituation, with particular regard to the sections concerning hygiene. Latrines should be available at camps and constructed at appropriate distances from water sources. If the staff or tourists have to defecate in the forest, faeces and even urine (as microsporidia can be transmitted also via urine) [61] must be buried properly in a 30 cm hole. To minimize the risk of transmission between humans and great apes, local staff health programs should be in place where increased human-gorilla contact occurs. Long-term and regular monitoring of great apes, humans and other wildlife will be essential in order to understand transmission patterns of the studied pathogens.
